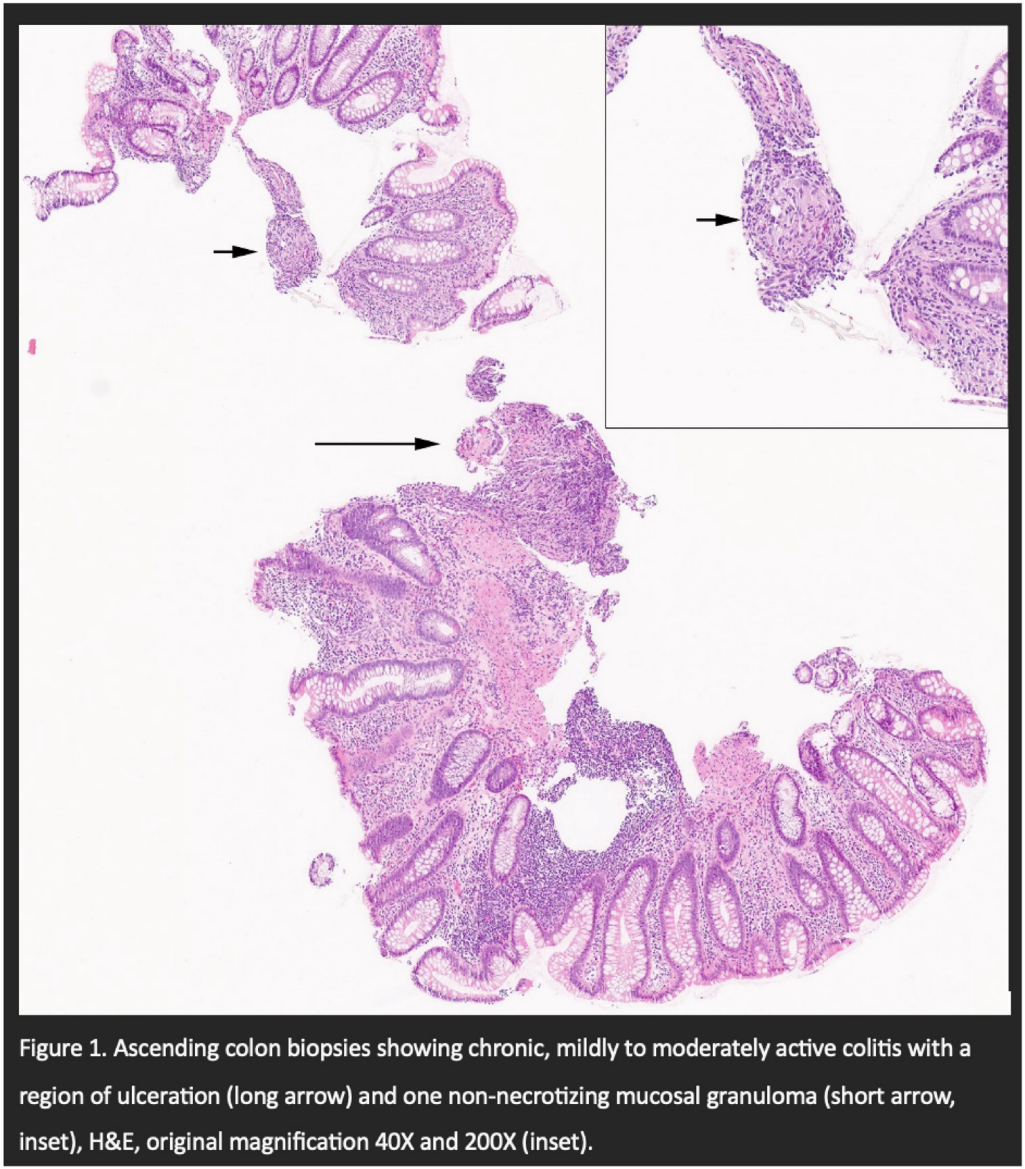# Poster Session I - A131 HYDROXYUREA-INDUCED ILEOCOLONIC ULCERATION: A CROHN’S DISEASE MIMICKER

**DOI:** 10.1093/jcag/gwaf042.131

**Published:** 2026-02-13

**Authors:** B R Tam, K Zhu, S Moosavi

**Affiliations:** Gastroenterology, The University of British Columbia Faculty of Medicine, Vancouver, BC, Canada; The University of British Columbia Faculty of Medicine, Vancouver, BC, Canada; Gastroenterology, The University of British Columbia Faculty of Medicine, Vancouver, BC, Canada

## Abstract

**Background:**

Hydroxyurea (HU) is a common cytoreductive therapy used in many hematological conditions. A rare association that has been documented with HU is cutaneous side effects including widespread skin changes and oral ulcers. The formation of non-oral gastrointestinal ulcers as a complication from HU is extremely rare and has been documented in only 4 cases reports.

**Aims:**

Case presentation

**Methods:**

58-year-old Indonesian male with a history of polycythemia vera on HU and aspirin for nearly 7 years presented with a positive FIT in 2022. Past medical history includes a ruptured appendix requiring an open appendectomy in 2019. He was asymptomatic with no side effects from HU therapy. He had no family history of gastrointestinal (GI) malignancies or inflammatory bowel disease. He had no tuberculosis risk factors and no regular alcohol or smoking history. A colonoscopy in Jan 2023 revealed terminal ileum and ascending colon ulcers on colonoscopy with pathology showing mild to moderately active inflammation with one non-necrotizing granuloma. A repeat colonoscopy 1 year after discontinuation of HU demonstrated complete resolution of the ileocolonic ulcers.

**Results:**

Pathophysiology of GI ulceration secondary to HU therapy is poorly understood. It is thought that HU-related mucocutaneous ulcers develop from the cumulative effects of direct cytologic damage to keratinocytes, from reduced blood flow and from impaired wound healing. Non-oral GI ulcers have been postulated to occur due to HU-induced hypersensitivity vasculitis or HU-induced Behcet’s disease. Only 4 documented case series have been published regarding HU-induced GI tract ulceration with 3 showing severe complications including significant GI bleeding, requiring surgical intervention. In all 4-case series, there was improvement and no recurrence after discontinuation of HU therapy, suggesting a causal relationship between non-oral GI ulcer and HU therapy. In this clinical case, he presented with an asymptomatic positive FIT test, highlighting a significant gap in our understanding of pathophysiology and prognostic factors in non-oral GI ulcers induced by HU.

**Conclusions:**

1) Early detection in HU induced colonic ulcers is critical in the prevention of severe complications such as massive GI bleeding or bowel perforation. 2) The need for strategies in the prevention and screening for potential life-threatening complications of HU induced GI tract ulceration. 3) Further research needs to be conducted in understanding the pathophysiology of GI ulceration within the GI tract.

**Funding Agencies:**

CAG